# The Role of Compassion in Altruistic Helping and Punishment Behavior

**DOI:** 10.1371/journal.pone.0143794

**Published:** 2015-12-10

**Authors:** Helen Y. Weng, Andrew S. Fox, Heather C. Hessenthaler, Diane E. Stodola, Richard J. Davidson

**Affiliations:** 1 Osher Center for Integrative Medicine, University of California, San Francisco, San Francisco, CA, United States of America; 2 Center for Investigating Healthy Minds at the Waisman Center, University of Wisconsin-Madison, Madison, WI, United States of America; 3 Waisman Laboratory for Brain Imaging and Behavior, University of Wisconsin-Madison, Madison, WI, United States of America; 4 Department of Psychiatry, University of Wisconsin-Madison, Madison, WI, United States of America; 5 Department of Psychology, University of Wisconsin-Madison, Madison, WI, United States of America; Middlesex University London, UNITED KINGDOM

## Abstract

Compassion, the emotional response of caring for another who is suffering and that results in motivation to relieve suffering, is thought to be an emotional antecedent to altruistic behavior. However, it remains unclear whether compassion enhances altruistic behavior in a uniform way or is specific to sub-types of behavior such as altruistic helping of a victim or altruistic punishment of a transgressor. We investigated the relationship between compassion and subtypes of altruistic behavior using third-party paradigms where participants 1) witnessed an unfair economic exchange between a transgressor and a victim, and 2) had the opportunity to either spend personal funds to either economically a) help the victim or b) punish the transgressor. In Study 1, we examined whether individual differences in self-reported empathic concern (the emotional component of compassion) was associated with greater altruistic helping or punishment behavior in two independent samples. For participants who witnessed an unfair transaction, trait empathic concern was associated with greater helping of a victim and had no relationship to punishment. However, in those who decided to punish the transgressor, participants who reported greater empathic concern decided to punish less. In Study 2, we directly enhanced compassion using short-term online compassion meditation training to examine whether altruistic helping and punishment were increased after two weeks of training. Compared to an active reappraisal training control group, the compassion training group gave more to help the victim and did not differ in punishment of the transgressor. Together, these two studies suggest that compassion is related to greater altruistic helping of victims and is not associated with or may mitigate altruistic punishment of transgressors.

## General Introduction

Cultivating altruistic and cooperative behavior among diverse individuals and communities is a challenge for our increasingly interconnected society. A growing body of literature is demonstrating that compassion meditation training can promote altruistic behavior among strangers [1–3]. Compassion is thought to increase altruistic behavior through the emotional response of caring for and wanting to relieve suffering [4–6], and compassion meditation trainings aim to cultivate responses of compassion when encountering suffering through contemplative practices and cognitive restructuring [7–9]. Little is known whether compassion training uniformly enhance many types of altruistic behavior or may be targeting specific kinds of altruism such as helping or punishment. Contemplative traditions from which many compassion trainings are derived posit that any action can be motivated by compassion [10]. Both basic science and intervention studies of compassion have mostly investigated the impact on altruistic helping behaviors towards victims [1,2,4,6], but less is known about the impact of compassion training on altruistic punishment of transgressors. It is important to identify which types of altruistic behavior compassion training can impact in order to optimally administer and implement trainings in targeted and context-appropriate ways.

Enhancing altruism has many potential benefits because altruistic behavior has positive effects on mental and physical health [11–14] as well as economic and societal growth [15,16]. Altruistic behavior is inherently social and involves an individual using personal resources to impact another individual or group [11,16]. Personal resources given to those in need can include instrumental social support (e.g., financial assistance), informational social support (e.g., providing information to help another cope), and emotional social support (e.g., expressing empathy and caring) [11]. Social support may benefit both the giver and receiver. Individuals who give instrumental social support show a decreased mortality rate, even when controlling for demographic, personality, health, mental health, and marital-relationship variables [17]. People report feeling more happiness after spending money for someone else rather than themselves [12]. Individuals who receive social support show improved mental and physical health (such as decreased depression and improved immune functioning) because they are more protected from the negative effects of stress [11,14].

Societal benefits may also occur through altruistic behavior that involves punishment, where personal resources are used to negatively impact those who have transgressed against others. Altruistic punishment may be a mechanism through which social norms, or what constitutes appropriate behavior within groups, are enforced [15,16,18]. Social norms aim to foster social peace, stabilize cooperation and enhance prosperity [15], and violation of cooperation and egalitarian distribution norms evoke altruistic punishment behaviors [16,18,19]. In a public goods game, the introduction of altruistic punishment of defectors increases cooperation over repeated interactions where the lack of punishment leads to less cooperation over time [18]. Those who are future-oriented in a delay discounting task and punish in the Ultimatum Game may be punishing to enforce transgressors’ future cooperative behavior [20]. People are motivated to punish players who make unfair offers towards themselves [16,18] as well as others (called “third party punishment” [16,19]), even at a personal cost. Further suggesting that altruistic punishment may serve to enforce social norms, punishment increases as unfair offers increase [18,19], and when unfair offers are rejected in the Ultimatum Game, proposers make more fair offers in subsequent rounds of play [16]. These results suggest that people are motivated to altruistically punish to enforce social norms such as cooperation and fairness, and that punishment does indeed result in more prosocial actions in those who were punished. Punishment may also serve antisocial motives such as competitive spite [21,22], and prosocial motives of punishment may be distinguished from antisocial motives through observing fairness of behavior played in other roles (such as the dictator in Dictator Game [21] and the proposer in the Ultimatum Game [22]) or punishment of cooperative players [23,24]. Prosocially-motivated punishment in this way can be viewed as an altruistic behavior that ultimately helps others.

In order to develop interventions that cultivate prosocial behaviors, it is important to understand both the emotions that lead to different types of altruistic behavior and the contextual factors that will promote the emotions. Typically, emotions such as empathy and compassion have been studied as antecedents of helping behavior [4,5,25], and negative emotions such as anger and annoyance has been studied as antecedents of punishment behavior [18,19]. However, compassion may be theoretically linked to both altruistic helping and punishment. Compassion as a naturally occurring emotion has been defined as having both an emotional and motivational component when encountering another person suffer, where the emotional component is one of caring and concern for someone’s suffering (also called “empathic concern”), and the motivational component includes the desire to help relieve suffering [6]. From this definition, it is clear how compassion would lead to altruistic helping of a victim. Indeed, studies show a link between the emotional state of compassion and the subsequent reported intentions of helping [25] or actual helping behavior [25].

Although it is less conceptually clear that compassion would influence altruistic punishment, there are several reasons why compassion may impact punishment. First, contemplative traditions emphasize that the motivation of the action is what is important rather than the action itself [10]. This suggests that punishment could be motivated by compassion to help the victim, the perpetrator, or both. If transgressors who violate fairness norms are financially punished, they are less likely to transgress in future interactions [16]. By deciding to punish, third parties may help protect future potential victims and also provide valuable feedback to the transgressors regarding the social acceptability of their behavior. Alternatively, if the transgressor is also viewed as a person who is suffering, compassion may lead to less punishment and other pathways to responding to the transgressor may emerge, as is seen in restorative justice systems [26]. One study showed that inducing compassion towards one stranger is associated with decreased punishment towards a separate transgressor [27]; however, it is unclear whether compassion towards the transgressor would yield a similar result, and punishment was not costly. Another study demonstrated that greater trait levels of the emotional component of compassion (empathic concern [28]) predicted greater costly altruistic redistribution of funds from a dictator who shared an unfair amount of money to the anonymous victim [3]. However, in this study altruistic helping of the victim and punishment of the transgressor were confounded, so it is unclear whether trait empathic concern motivated helping, punishment, or both.

To clarify this issue, we tested the relationship between compassion and altruistic helping and punishment behavior in two studies. In Study 1, we tested whether the emotional component of compassion (measured by self-reported trait empathic concern) is associated with altruistic helping and/or punishment behavior in response to an unfair economic exchange. In Study 2, we tested whether direct enhancement of compassion through short-term compassion training would impact altruistic helping and punishment behavior measured after training.

To measure altruistic behavior, we used paradigms from behavioral economics because they provide powerful tools to study instrumental social support [29]. Compassion and altruistic behavior are socially desirable qualities, and they are highly susceptible to demand characteristics [3,30]. In addition, participants in social psychological studies of compassion and helping behavior often report a desire to help but not are required to act on that help at the conclusion of the experiment [4]. Using behavioral economic paradigms address some of these methodological concerns by studying social behavior through computerized anonymous one-shot interactions [29]. This minimizes the effects of demand characteristics and reputation effects because participants play with live players in the laboratory only once and do not know which players they are interacting with at any given time. Furthermore, any economic decisions participants make directly impact their monetary compensation, decreasing the likelihood they are acting solely to maintain their reputation in front of peers or experimenters. Economic paradigms may have less ecological validity compared to real-world social interactions due to these design elements; however, this tightly-controlled method of specifying and measuring social behavior allows us to make more concrete conclusions about the relationship between emotional traits and types of altruistic behavior.

We used third-party altruistic paradigms [19] to measure altruistic punishment and helping behavior. Third-party paradigms are useful for studying compassionately-motivated behavior because they involve 1) witnessing an unfair economic interaction between a dictator and a victim (a model of “suffering” or unfair treatment) and 2) the opportunity for the participant to altruistically spend personal funds to impact another player such as punishing the dictator [19], helping the victim, or redistributing funds from the dictator to the victim [3]. In Study 1, we administered the third-party altruistic helping and punishment paradigms in two separate samples, and determined the extent to which the emotional component of compassion (self-reported trait empathic concern) was associated with altruistic behavior in the games. In Study 2, we tested whether direct enhancement of compassion through short-term compassion training would increase altruistic helping and/or punishment behavior compared to a cognitive reappraisal control group.

Compassion meditation training stems from Buddhist philosophy and contemplative traditions, and some Buddhist conceptualizations of compassion are in alignment with the Western psychological definition stated earlier [6], where it is described as being moved by another’s suffering and resulting in a desire to relieve the person’s suffering [7,9] (however, see [31] for an alternate conceptualization of compassion which views it as dispositionally enactive and as a contingent and emergent process). Compassion meditation practices aim to enhance compassionate responses towards the suffering of the self and others using a variety of techniques such as visualization practices, repeating compassion-generating phrases (such as, “May you be free from suffering” and “May you be safe”), bodily awareness, and cognitive education in Buddhist philosophy [2,9,32,33]. Compassion may be generated towards different people such as a loved one, the self, a stranger, and a “difficult” person with whom there is conflict, and towards all people [9]. Compassion training typically trains balanced awareness to stimuli of suffering, enhancing emotional compassionate feelings towards those who are suffering, and enhancing the motivation to relieve suffering [2,3,9,33]. These practices may be conducted while in a sitting position as well as during walking meditation (e.g., offering wishes and phrases of compassion for others while walking) [2,9]. It is worth noting that within the context of altruistic punishment, generating compassion for a “difficult person” is conceptually aligned with practicing compassion for a transgressor.

Studies of compassion training have mostly investigated the relationship between compassion and altruistic helping behavior. A one-day compassion training (6 hours total) conducted with 28 women resulted in greater helping behavior in the Zurich Prosocial Game compared to 32 women in a memory training group [2]. From pre- to post-training, the compassion-trained group were more helpful to another player in the computerized Zurich Prosocial Game compared to the control group, both when it was costly and non-costly for themselves [2]. In another study, both 8 weeks of compassion (Cognitively-Based Compassion Training; 16 hours total) and mindfulness training (meditation practices that trained nonjudgmental awareness without a focus on compassion) resulted in greater prosocial behavior towards an injured confederate stranger compared to a wait-list control [27].

Some researchers theorize that compassion should enhance cooperative behaviors whereas fairness-based norms should motivate punishment, and acknowledge that empirical research is needed to test these hypotheses [34]. Our group found that short-term compassion training (7 hours total over 2 weeks) increases third-party altruistic redistribution, which is a combination of both third-party punishment and helping [3]. However, because the two behaviors were combined, it is unclear whether compassion influences the punishment or helping components of third-party altruism or both. Long-term practitioners of compassion meditation were found to punish less when given an unfair split themselves, and punished equivalently when viewing another receive an unfair split compared to a control group [35]. They were also given the opportunity to recompensate the victim who received the unfair split, and spent more to aid the victim compared to the control group [35]. These findings suggest that compassion may be less likely to impact (or may even mitigate) retributive punishment behavior and may enhance helping behaviors that restore equity. However, due to the cross-sectional design using long-term practitioners, differences in behavior cannot be purely isolated to compassion meditation practice and may be due to other lifestyle factors such as other contemplative practices or living in a retreat setting. In Study 2, we directly trained healthy participants in compassion using a randomized control design in order to make stronger claims about the relationship between compassion and altruistic helping and punishment behaviors.

In summary, we investigated the relationship between compassion and altruistic helping and punishment using third-party economic paradigms by testing the relationships 1) as they naturally occur in the general population and 2) by directly influencing compassion through compassion training. In Study 1, we recruited participants from the general population to investigate whether trait empathic concern (the emotional component of compassion) was associated with third-party altruistic helping or punishment after witnessing an unfair or fair interaction. We also tested whether altruistic behavior was associated with trait negative affect. In Study 2, we investigated whether directly enhancing compassion through short-term online compassion training [3] would increase altruistic punishment or helping compared to a cognitive reappraisal control group. These games were administered in the same population as a previous study of compassion training that found increases in altruistic redistribution and neural responses to human suffering [3].

## Study 1: Individual Differences in Empathic Concern Associated with Altruistic Helping and Punishment

### Materials and Methods

#### Ethics Statement

The experiment was approved by the University of Wisconsin-Madison Social and Behavioral Sciences Institutional Review Board (Protocol SE-2009-0499). All participants were adults who provided informed consent and were paid based on their decisions in the economic games or at least $10/hour. No minors/children were recruited for the study. Participant consent was documented by study personnel, and signed copies of the consent forms were kept in secure locked files. The IRB approved this consent procedure.

#### Participants

All participants were adults recruited from the community of Madison, WI, United States of America. Independent samples were recruited for the Punishment Game and the Helping Game. In the Punishment Game, 143 participants were recruited, and 132 participants produced useable data (50 male; 82 female; mean age = 23.5 [*SD* = 8.4]). In the Helping Game, 139 participants were recruited, and 136 produced useable data (54 male; 82 female; mean age = 23.2 [*SD* = 5.5]).

#### Procedure

Participants were brought to the computer laboratory in groups (n = 9 or 12), and read the instructions on the game website. Experimenters confirmed that they understood the rules of the game, and then three rounds of the game were played. Participants used a web interface to ensure that each game interaction was played 1) with live players 2) anonymously and 3) with unique participants. This design allowed for real-time interactions with live players while minimizing reputation effects. To maximize data points, each participant played in each role (dictator, recipient, third party) with the order randomized. Participants were free to choose any decision in each position, and no deception was used. Payment was determined by game outcome. Trait questionnaires were completed either before or after game playing.

#### Measures

**Fig 1:**
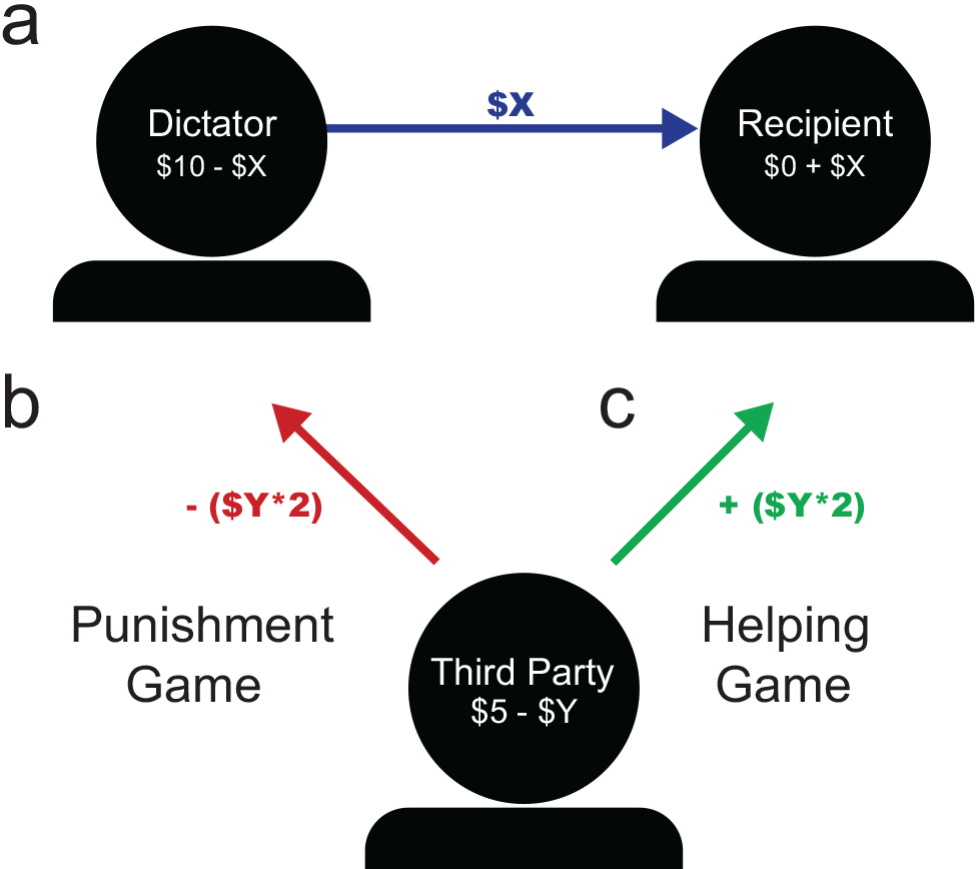
Third-party punishment and helping game paradigms. **a)** In the first step of the games, the Dictator transfers any X amount of $10 (100 points) to the anonymous Recipient while the Third Party observes. **b)** In the Punishment Game, the Third Party may spend any Y amount of $5 (50 points) to take twice the amount from the dictator, constrained by the amount the dictator originally gave (cannot punish below $0). **c)** In the Helping Game, the Third Party may spend any Y amount of $5 (50 points) to transfer twice the amount to the Recipient. In the Compassion and Reappraisal Training study, all participants witnessed an unfair Dictator transfer (< $2.50/$10).

To measure altruistic behavior, third-party economic decision-making paradigms were used (Fig 1). All the games involved three players (the dictator, recipient, and third party) and two interactions in the game. The games each began with an interaction between the dictator and the recipient, but differed in how the third party could impact the other players. In each game, the dictator was endowed with 100 points, a recipient with 0 points, and a third party (the participant of interest) with 50 points. In the first interaction of the game, the dictator may choose to transfer any number of the 100 points to the recipient, while the third party observes (Fig 1A). The third party can then respond based on the rules of the game (see below). In the online game, the roles are described with neutral language where the dictator is labeled as “Participant 1”, the recipient is labeled as “Participant 2”, and the third party is labeled as “Participant 3”

When the game is over, points are converted to dollars (10 points = $1), and each player is paid based on the number of points acquired. Therefore, decisions directly affect monetary outcomes. Participants were paid at least $10/hour.

#### Punishment Game

Similarly to Fehr & Fischbacher [19], in the second interaction, the third party may choose to spend points in order to take points away from the dictator. Each point spent by the participant results in two points deducted from the dictator (Fig 1B). Participants can punish any amount without exceeding the value of the dictator’s remaining points after transferring to the recipient.

#### Helping Game

In the second interaction, the participant may choose to spend points in order to transfer points to the recipient. Each point spent by the participant results in two points given to the recipient (Fig 1C).

#### Questionnaires

To assess whether altruistic behavior is associated with reported trait levels of compassion, participants completed the Empathic Concern subscale of the Interpersonal Reactivity Index, which measures the emotional component of compassion and is described as “the tendency to feel warmth, compassion and concern for others undergoing negative experiences [28]”. Participants rated items such as “I often have tender, concerned feelings for people less fortunate than me” on a scale of 0 (does not describe me very well) to 4 (describes me very well). Although the term “compassion” is used within the definition of empathic concern [28], upon closer examination of the items, the subscale only measures the emotional component of compassion (caring and concern) and does not directly assess the desire to help people who are suffering.

The Empathic Concern scale was chosen because in our previous study of associating trait empathic concern with altruistic redistribution behavior [3], empathic concern showed the strongest relationship with redistribution compared to other measures of compassion such as the Compassionate Love Scale (CLS [36]) and the Compassion subscale of the Dispositional Positive Emotions Scale (DPES [37]). The Compassionate Love Scale for strangers and humanity measures a more enduring and encompassing state that may contribute to sustained prosocial behavior [36] and directly assesses the desire to help (e.g., “If I encounter a stranger who needs help, I would do almost anything to help him or her”). The Compassion subscale of the DPES also measures the motivation to help (“take care of”) people who are vulnerable as well as the tendency to notice people who need help and valuing helping them [37]. All three scales were highly intercorrelated in a previous study associating trait compassion with altruistic redistribution behavior (all *r*’s > 0.74 [3]), which suggests that subcomponents of compassion such as empathic concern and the desire to help are strongly related. We decided to only administer the Empathic Concern scale in these studies because it showed the strongest correlation with redistribution, it was highly correlated with other scales of compassion, and to save participant time; however, we acknowledge the motivational component of compassion was not directly measured and should be incorporated in future studies.

Trait negative affect was administered using the Positive and Negative Affective Scales [38], and items such as “distressed” and “hostile” were rated on a scale from 1 (very slightly or not at all) to 5 (extremely). To control for possible confounds of social desirability and current mood, the Marlowe-Crowne Social Desirability scale [30] and state Positive and Negative Affective Scales [38] were administered. Family income was also measured using a scale that measured income from $10,000 to > $200,000.

### Data Analysis

#### Data reduction

In the Helping Game, 142 participants were recruited, and 139 produced useable data. Three data points were excluded because of game website errors. See data in S1 Dataset. Three participants were identified as group outliers due to being > 3 *SD* above the mean in helping percentage (N = 2) or > 3 *SD* below the mean in trait empathic concern within participants who witnessed an unfair interaction (N = 92). The three group outliers were also considered highly influential points in a regression of empathic concern predicting helping behavior in response to unfair exchanges, and scored above the empathic concern DFBETA cutoff of 0.281 (computed by 2/sqrt(N = 92); the DFBETA is a measure of how much an observation has affected the estimate of a regression coefficient), therefore we report the main findings without these 3 outliers (final N = 136; unfair N = 89).

Upon closer inspection of the outliers, the two helping percentage outliers gave their entire endowment as the third party after viewing an unfair dictator offer (all 50 points). In addition, these 2 participants also gave their entire endowment when they themselves played as the dictator. These participants may be considered “extreme altruists” and give their entire endowment independent of the social context, and they may be behaving from different motives compared to the rest of the group. We consider these “extreme altruists” interesting and worthy of study in further studies, so we also report results from additional analyses that includes these 2 group outliers in Study 1 (N = 138). The empathic concern outlier was the most highly influential outlier who reported no levels of empathic concern (score of 0), yet gave 50% of their third-party endowment in response to an unfair dictator transfer. We consider reporting no empathic concern highly unusual and consider this participant a true outlier. This participant may have been acting more from fairness-based norms rather than compassion-based norms [34] which differentiated them from the rest of the sample. The final sample included 136 participants (54 males, 82 females, average age = 23.2 [*SD* = 5.5]). The supplementary sample with the two helping game outliers included 138 participants (54 males, 84 females).

In the Punishment Game, 143 participants were recruited, and 132 participants produced useable data. 9 data points were excluded because of game website errors, and an additional 2 participants were not included in data analyses because they were not able to make decisions after the dictator gave the full 100 points to the recipient. The final sample included 50 males and 82 females, with an average age of 23.5 (*SD* = 8.4).

Altruistic behavior was calculated as a percentage of the total possible altruistic amount, where the raw number of points was divided by the maximum points that could be spent. The percentage metric was chosen because in the games that included punishment, altruistic behavior was constrained by the remaining dictator endowment after transferring to the recipient (see [3] for full rationale of the percentage metric). A punishment score of 50%, for example, could represent spending 50/100 points as well as 40/80 points. Participants who played the Helping Game could spend up to the full 50 points to help the recipient independent of the dictator’s choice. Because compassion is evoked by unfair treatment [6], we analyzed participants who witnessed an unfair dictator transfer (≤ 25%) separately from participants who witnessed a fair or generous dictator transfer (≥ 50%). Unfair offers have been similarly defined in other economic decision-making studies [39,40].

#### Individual differences with trait empathic concern

To test whether trait empathic concern is associated with altruistic punishment and/or altruistic helping, we correlated self-reported empathic concern with behavior in the two samples in both the unfair (dictator spends ≤ 25%) and fair/generous conditions (dictator spends ≥ 50%). As an exploratory test within the altruistic punishment game, we also correlated trait compassion with punishment behavior in the participants who decided to punish at all (called the Punishers, spent > 0 points). We further split the sample into Prosocial Punishers and Antisocial Punishers based on their behavior as the dictator [21], and examined whether trait empathic concern was correlated with punishment in each group. Prosocial Punishers were defined as those who behaved fairly/generously when playing as the dictator (spent ≥ 50%), and they may punish based on prosocial motives of enforcing the fairness norm and therefore show a positive correlation between empathic concern and punishment. Antisocial Punishers were defined as those who behaved unfairly when playing as the dictator (spent ≤ 25%), and they may punish based on antisocial motives (such as anger or envy) and show a negative correlation between empathic concern and punishment.

#### Controlling for confounding variables

The third-party responses could possibly be influenced by confounding variables of social desirability, general prosocial behavior (as indicated by transfers made when participants played as the dictator), family income, previous earnings or punishment in the game before playing as the third party, and current mood (state positive or negative affect). To determine that compassion predicts altruistic behavior over and above the confounding variables, we used hierarchical linear regression modeling. On the first step, we entered measures of social desirability, general prosocial behavior (behavior when the participant played as the dictator), family income, previous experience in the game (total earnings and punishment percentage before playing as the third party), and current positive and negative affect. On the second step, we entered trait empathic concern. In the Punishment Game, due to the relationship found within the previous zero-order correlation test, this test was performed within the Punishers sub-group (spent > 0 points) only.

#### Comparing empathic concern-behavior relationships between games

After determining the relationship with empathic concern in the Punishment and Helping Games, we tested whether the relationships were significantly different from each other using the Fisher r-to-z transformation as well as a hierarchical linear regression model that controlled for significant confounding factors. Because a relationship with empathic concern was found within the Punishers only, these tests were only performed in the Punishers. Furthermore, we wanted to determine whether the relationship with compassion in the Redistribution Game significantly differed from the relationship in the Helping and Punishment Games, so these tests were also performed with Redistribution Game data previously reported in the supplemental material in [3].

The difference between the zero-order correlations in each game pair (Helping vs. Punishers, Redistribution vs. Helping, Redistribution vs. Punishers) was calculated using a Fisher r-to-z transformation. Within the hierarchical regression models, the first step included significant confounding factors identified in the first regression model for the relevant games, as well as all potential Game × Confounding Factor interactions (e.g., social desirability is significantly associated with Redistribution but not Helping behavior, so a Game × Social Desirability interaction term is modeled). Main effects of Game and Empathic Concern were also entered in the first step. To identify unique variance associated with differences in the compassion-altruistic behavior association between games, the Game × Empathic Concern interaction term was entered in the second step.

#### Individual differences in negative affect

To investigate whether individual differences in negative emotions are associated with altruistic behavior, we correlated trait negative affect [38] with altruistic behavior in each game in fair/generous and unfair conditions. To examine the relationship between negative affect and altruistic behavior that includes both punishment and helping behavior, we also performed a novel correlation test to see if trait negative affect is associated with redistribution behavior in the sample previously reported in [3].

### Results

#### Individual differences in empathic concern and altruistic behavior

**Fig 2:**
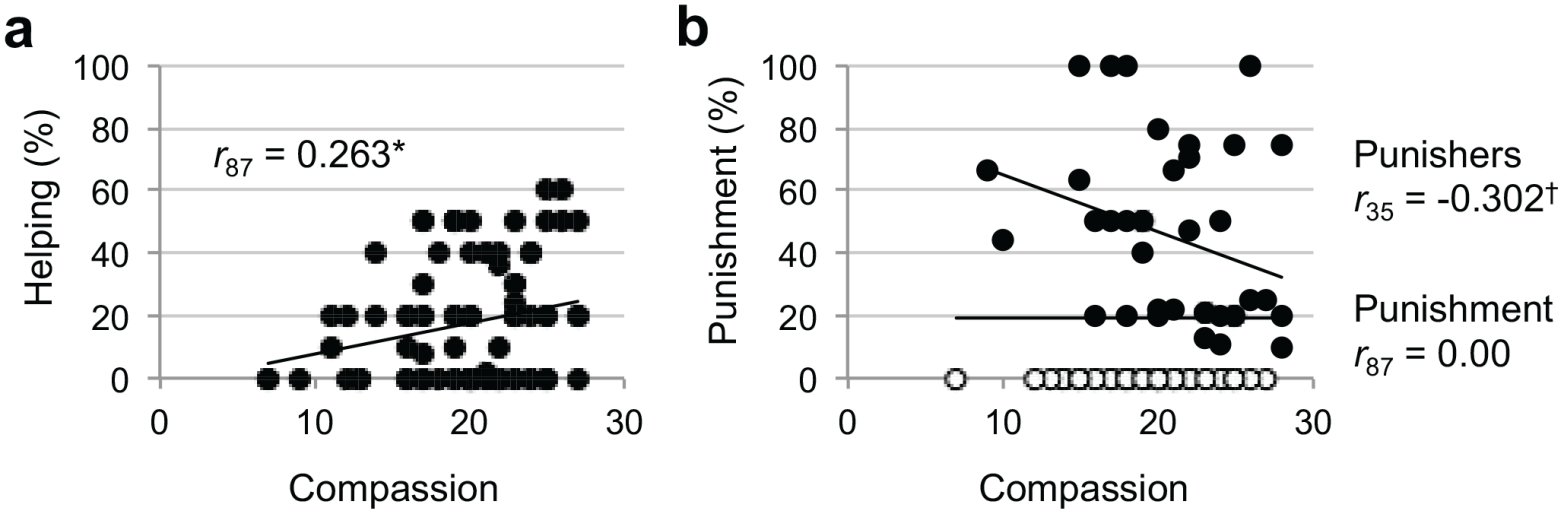
The association between trait compassion and third-party altruistic behavior after an unfair dictator transfer. **a)** In the Helping Game, individuals who report greater compassion give more to the recipient after an unfair interaction (< = 25%). Including the two “extreme altruist” outliers in Helping Game responses, the correlation remains significant (*r*_89_ = 0.214, *p* < 0.05). **b)** In the Punishment Game, trait compassion is not associated with punishment behavior after an unfair interaction in the full sample. However, within Punishers (individuals who decided to punish at all and spend > $0, indicated by black shaded circles), those who report greater compassion decide to punish less at trend level. ^†^ *p* < 0.1, * *p* < 0.05

As hypothesized, participants who reported greater trait empathic concern gave more in the Helping Game after witnessing an unfair dictator transfer (*r*_87_ = 0.236, *p* < 0.01, Fig 2A; when including outliers *r*_89_ = 0.214, *p* < 0.05). There was no relationship between trait empathic concern and punishment behavior (*r*_87_ = 0.00, *p* = 1, Fig 2B). However, when inspecting the participants who punished at all (Punishers, spent > $0; N = 37), the relationship between empathic concern and punishment was marginally negative (Punishers *r*_35_ = -0.302, *p* < 0.1, Fig 2B). This relationship is driven by the Antisocial Punishers, who played unfairly as the dictator and punished as the third party (*r*_19_ = -.40, *p* = .07; relationship was not significant including participants who were unfair as the dictator and did not punish [spent $0], *r*_56_ = .070, *p* = 0.60). Prosocial Punishers, who played fairly/generously as the dictator and punished as the third party, did not show a significant correlation between empathic concern and punishment (*r*_8_ = -0.16, *p* = 0.66; including those who did not punish, *r*_15_ = -0.10, p = 0.70). Trait empathic concern did not predict altruistic behavior in any game in response to fair/generous dictator transfers (all *p*’s > .63).

In the unfair condition, greater empathic concern was still associated with altruistic helping and punishment (punishers only), even after controlling for potentially confounding variables of social desirability, behavior when playing as the dictator, family income, player order, previous earnings and punishment before playing as the third party, and current positive and negative affect (Tables 1 and 2). Individuals who reported greater compassion spent more in the helping game and spent less in the punishment game if they decided to punish at all (all *p*’s < 0.05; Tables 1 and 2).

**Table 1:** Trait empathic concern predicts altruistic behavior after an unfair dictator transfer even after controlling for confounding variables in a hierarchical regression model. Step 1 modeled potential confounding variables of social desirability, general prosocial behavior (as indicated by transfers made when participants played as the dictator), family income, previous earnings or punishment in the game before playing as the third party, and current mood (state positive or negative affect). Trait empathic concern (the emotional component of compassion) was entered in Step 2 and was measured by the Empathic Concern subscale of the Interpersonal Reactivity Index (Davis, 1980). Punishers represent a subset of participants who played the Punishment Game that decided to punish by spending > $0 (N = 37/89).

	*Game* Helping	Punishers
N (Unfair)	89	37
Step 1: Confounds Δ *R* ^2^	0.31***	0.22
Step 2: Empathic Concern Δ *R* ^2^	0.04* ^	0.18**

* *p* ≤ 0.05

** *p* ≤ 0.01

*** *p* ≤ 0.001

^ When the two “extreme altruist” outliers were included in the Helping Game regression, the relationship between empathic concern and helping behavior became non-significant (Empathic Concern Δ *R* ^2^ = 0.016, *p* = 0.12).

**Table 2:** Semipartial correlations indicating the unique variance predicted by each variable in the full hierarchical regression model. Trait empathic concern (the emotional component of compassion) predicts unique variance in both the Helping Game and within Punishers (spend > $0) in the Punishment Game.

	*Game*	
	Helping *sr*	Punishers *sr*
Social Desirability	-0.11	0.42**
Transfer as Dictator	0.46***	0.21
Player Order	-0.06	0.04
Family Income	-0.01	0.01
Experience in game: Punishment		0.04
Experience in game: Earnings	0.07	0.06
Current Positive Affect	0.07	-0.15
Current Negative Affect	-0.01	0.04
Empathic Concern	0.19* ^	-0.42**

* *p* ≤ 0.05

** *p* ≤ 0.01

*** *p* ≤ 0.001

^ When the two “extreme altruist” outliers were included in the Helping Game regression, the relationship between empathic concern and helping behavior became non-significant (Empathic Concern Δ *R* ^2^ = 0.016, *p* = 0.12).

#### Game differences in empathic concern-altruism relationships

In the unfair context, the positive relationship between empathic concern and helping behavior was significantly different from the negative relationship between empathic concern and punishment behavior in Punishers (*Z* = 2.87, *p* < 0.01; including helping game outliers *Z* = 2.62, *p* < .01). In terms of the strength of the empathic concern-behavior relationship, the redistribution game demonstrated similar patterns as the helping game. The empathic concern-redistribution correlation did not significantly differ from the empathic concern-helping correlation (*Z* = 1.19, *p* = 0.23; including helping game outliers *Z* = 1.53, *p* = 0.13), but was significantly greater than the empathic concern-punishment correlation in Punishers (*Z* = 3.69, *p* < 0.001).

This pattern of results was confirmed even when controlling for confounding factors. The positive association between empathic concern and helping behavior was greater than the negative association between empathic concern and punishment (Helping vs. Punishers interaction term *R* ^2^ change = 0.06, *F*_1,99_ = 14.71, *p* < 0.001). In addition, the positive relationships that empathic concern demonstrates with helping and redistribution behavior did not significantly differ from each other after controlling for significant confounding factors (Redistribution vs. Helping interaction term *R* ^2^ change = 0.008, *F*_1,150_ = 1.82, *p* = 0.18; including helping game outliers *R* ^2^ change = .01, *F*_1,152_ = 2.65, *p* = 0.11). Redistribution was significantly more associated with empathic concern compared to punishment behavior (Redistribution vs. Punishers interaction term *R* ^2^ change = 0.13, *F*_1,99_ = 22.13, *p* < 0.001).

#### Individual differences in negative affect

Trait negative affect did not predict altruistic behavior in any game (Helping, Punishment, or Redistribution) when the dictator transferred an unfair amount (all *p*’s > 0.53). However, participants who altruistically punished or redistributed funds in response to a fair or generous dictator transfer reported more trait negative affect (Punishment *r*_23_ = 0.59, *p* < .01; Redistribution *r*_40_ = .45, *p* < 0.01). No relationship was found in the Helping Game.

#### Other predictors of altruistic behavior

In each game, one or more confounding factors also emerged as significant predictors of altruistic behavior in addition to trait compassion (see Table 2). Within Punishers in the Punishment Game, the amount of punishment was positively predicted by social desirability (*sr* = 0.42, *p* < 0.01), although this relationship does not hold true for the full punishment sample (*sr* = 0.13, *p* = 0.23). In the Helping Game, altruistic behavior was positively associated with the participant’s transfer when playing as the dictator (*sr* = 0.46, *p* < 0.001).

### Discussion

We found that the emotional component of compassion, or the tendency to feel warmth, caring, and concern for those who are suffering, does not uniformly impact altruistic behavior but is specific to promoting altruistic helping of a victim and not altruistic punishment of a transgressor. In the Helping Game, individuals who reported more trait empathic concern spent more funds to directly help the victim who was treated unfairly. In the Punishment Game, there was no relationship between empathic concern and punishment within the entire sample. However, in the participants who decided to punish at all (Punishers), the ones who endorsed greater empathic concern were the ones who punished the least. Furthermore, this relationship was driven by Antisocial Punishers, who behaved unfairly when playing as the dictator (within the context of the third-party punishment game) and punished unfair dictators themselves when playing as the third party. This set of findings suggest that general feelings of empathic concern towards those who are in need are indeed associated with acting on those feelings and helping someone who is treated unfairly. In contrast, general feelings of empathic concern do not appear to be related to punishment of a transgressor overall. However in those who decide to punish (particularly in those who demonstrate antisocial behavior as the dictator), trait empathic concern may mitigate the degree to which they punish, and this may balance competing motivations to discourage the transgressor from future violations of the fairness norm while not being overly punitive. This finding is similar to other studies that suggest that compassion decreases punishment when another [27] or the self [35] is transgressed. Future studies should examine whether compassion may be positively associated with punishment in larger samples of Prosocial Punishers, those who are prosocially-motivated as indicated by fair/generous behavior played in other roles. Prosocial and Antisocial Punishers can be more cleanly identified in future studies by administering the third-party punishment game in conjunction with the dictator game.

The emotional component of compassion may impact altruistic behavior that involves any component of helping, even if the helping behavior is coupled with punishment (as in the Redistribution Game). Currently, the data suggest that empathic concern impacts altruistic helping and redistribution similarly, but more data may be needed to detect statistical differences (the empathic concern-redistribution relationship was marginally significantly greater than the empathic concern-helping behavior relationship when the “extreme altruists” in the helping game were included). The helping and redistribution behaviors have fundamentally different economic and social outcomes. Redistribution impacts the transgressor while helping does not, and because it impacts both parties simultaneously, it is a behavioral representation of justice that has both a monetary and psychological impact. Redistribution mathematically decreases inequality between the dictator and recipient at twice the rate as helping or punishment, and further studies are needed to determine whether this difference impacts the relationship with compassion. In addition, for some participants, it may be psychologically desirable to impact both players after an unfair interaction in order to both help the victim as well as negatively reinforce the dictator to discourage future transgressions (and protect future victims).

Trait negative emotions did not impact altruistic helping, punishment, or redistribution behavior after an unfair transaction. This is somewhat counter to previous findings that negative emotions such as anger positively predict altruistic punishment [19,35,41]. However, negative emotions were measured at the trait rather than state level, and the measure assessed many different types of negative emotions rather than isolating specific states that may be more associated with punishment (such as anger and annoyance). Interestingly, trait negative emotions did positively predict greater punishment and redistribution after a fair or generous dictator transfer. It is surprising that participants would be motivated to spend personal funds to punish a stranger who acted fairly because it is economically costly. Previous research has shown that few people punish after a fair split and most participants do not *believe* players will punish in that case [19], although antisocial punishment of prosocial players varies widely across societies [23]. Participants may receive other psychological benefits from antisocial punishment that justifies the expense, and this may be associated with experiencing negative emotions on a regular basis.

This study found that other factors may reliably predict altruistic behavior and should be taken into consideration in future studies. Economists state that decisions are not due to reputation effects in one-shot games because the interactions are anonymous and participants only interact once [29]. However, our data demonstrated that social desirability was a significant predictor of altruistic behavior in both Punishment and Redistribution Games, and previous work shows this effect is driven by interacting with other live players rather than playing in isolation [3]. Notably, only games that involved punishment of the dictator were associated with social desirability, and this suggests that it may be socially desirable to punish norm-violators rather than to help victims. Third-party behavior in the Helping Game was highly correlated with participants’ behavior while playing as the Dictator. In fact, more unique variance in helping behavior was associated with behavior while playing as the Dictator than with trait compassion. This suggests that altruistic helping may largely reflect a general desire to be prosocial, independent of the context of first witnessing an unfair interaction.

Future work should also directly measure the motivational component of compassion (perhaps through the CLS or DPES) to determine whether the emotional and motivational components of compassion contribute unique variance to enacted altruistic behavior. It remains challenging to distinctly measure empathic concern and motivation to help, and motivation to help is often inferred from observable altruistic behavior. It is likely that some people may have the desire to help but may not act on that desire for a variety of reasons, and further research should understand how this disconnect between motivation and action may occur. Finally, it is unclear how behavior in controlled economic decision-making paradigms, which provide crisp models of measuring altruistic behavior, is related to altruistic behavior in real-world scenarios. More research is needed to understand the relationship between behavior in these games to behavior in everyday life (e.g., helping a friend or giving negative feedback to a coworker) in order to understand the applicability of results from these paradigms.

## Study 2: Compassion Training Study

### Materials and Methods

#### Ethics Statement

The training study was approved by the University of Wisconsin-Madison Health Sciences Institutional Review Board (Protocol H-2006-0107), and all participants were adults who provided written informed consent and were paid for their participation. No minors/children were recruited for the study. Participants in the training study were also consented separately for the behavioral study approved by the Social and Behavioral Sciences Institutional Review Board to enhance their perception that the training study and economic behavior study were separate protocols and unrelated. Participant consent was documented by study personnel, and signed copies of the consent forms were kept in secure locked files. The IRB approved this consent procedure. Participants were paid based on decisions made in the economic games in addition to the $165 earned from completing the training study. Participants who did not complete the entire protocol earned $8/hour for their time.

#### Participants

All participants were adults recruited from the community of Madison, WI, United States of America. The participants include the same participants from a previous study of compassion training and altruistic redistribution [3]. Participants were healthy adults who were 18–45 years of age, right-handed, and had no previous experience in meditation or cognitive-behavioral therapy. Because the participants were originally recruited for a functional magnetic resonance imaging (fMRI) study, participants were also screened out for MRI contraindications including claustrophobia and ferromagnetic implants. Participants were included for analysis if they completed the entire training protocol (3 laboratory visits and practicing at least 11/14 days), which resulted in 56 participants. The groups did not differ in age, gender, baseline trait compassion, or the amount of practice time they engaged in. The final sample for the altruistic punishment game consisted of 41 participants who believed that they were interacting with real players in the economic games, and no demographic or baseline compassion differences were found between the believers and non-believers of the paradigm [3]. The helping game was added later to the entire training protocol, so only 39 participants played the game, with a final N of 30 believing the paradigm (Compassion Training N = 13, Reappraisal Training N = 17).

#### Procedure

The procedure was completed with the same sample as our previous study of compassion training and redistribution behavior, and the compassion and reappraisal training methodology used is the same (see [3] for full methodological details). Briefly, 56 healthy adults completed a protocol where they were randomized to either Compassion Training (N = 28) or Reappraisal Training (N = 28), practiced their respective trainings through guided Internet instruction for 30 minutes/day for two weeks, and played the economic games at their post-training visit. They completed an fMRI protocol at both pre- and post-training visits, and the results are reported in [3].

The Compassion Training group practiced a meditation exercise where they increased feelings of compassion towards different kinds of people including a loved one, the self, a stranger, and a person with whom they had conflict [3,9]. Through guided audio instructions, the Compassion trainees practiced compassion for each person by 1) contemplating and envisioning their suffering and then 2) wishing them freedom from that suffering. They imagined a time each person had suffered (e.g., illness, relationship problem,), and were instructed to pay attention to the emotions and sensations this evoked (the emotional component of compassion such as warmth and empathic concern). They practiced wishing that the suffering were relieved (the motivational component of compassion) and repeated the phrases, “May you be free from this suffering. May you have joy and happiness.” They used visualization techniques such as envisioning a golden light extending from their heart to the other’s heart to ease their suffering, and were instructed to focus on bodily sensations especially around the heart. At the end of the meditation, compassion was extended towards all beings. The script was developed by Linda Wuestenberg, M.Ed., a Licensed Clinical Social worker (LCSW) and a Certified Substance Abuse Counselor (CSAC) who has 33 years of clinical practice and has practiced compassion meditation in the Drikung Kagyu tradition of Tibetan Buddhist meditation.

We designed an active control intervention of Reappraisal Training to rule out the possibility that any type of emotional training would result in similar effects as Compassion Training. The trainings were balanced in the time, effort, mode of administration, and expertise of the clinician (see [3] for full details). The Reappraisal Training group practiced reinterpreting personal stressful events to decrease negative emotions. The reappraisal script was modeled after homework exercises used in cognitive behavioral therapy [42]. They were asked to recall a stressful experience from the past 2 years that remained upsetting to them and to vividly recall details of the experience. They wrote a brief description of the event, described their emotions, and rated the intensity of the emotion both during the event and at the present moment. Then they were asked to reappraise the event (to think about it in a different, less upsetting way) using 3 different strategies, and to write down the new thoughts. The strategies included 1) thinking about the situation from another person’s perspective (e.g., friend, parent), 2) viewing it in a way where they would respond with very little emotion, and 3) imagining how they would view the situation if a year had passed, and they were doing very well. They completed emotion ratings after employing each reappraisal strategy. For example, if a participant was upset about a disagreement with a coworker, they could reappraise the situation by imagining that in a year, the disagreement would have been resolved and it would no longer be so distressing. The training was written by Gregory Rogers, Ph.D., a clinical psychologist who was licensed in 1999, certified as a cognitive therapist by the Academy of Cognitive Therapy, and is a member of the Association for Behavioral and Cognitive Therapies. Training audio files and written scripts can be downloaded at http://www.investigatinghealthyminds.org/compassion.html.

After the two weeks of training, they returned to the laboratory and played the economic games. To decrease demand characteristics, they were instructed that the economic paradigms were a separate study and asked to sign a separate IRB protocol. The economic games were only administered post-training to increase believability that the games were not tied to the compassion training protocol. In addition, it is unclear how one-shot economic decisions would function in a pre-post paradigm, so we only administered the games post-training. To account for the lack of baseline responses, we compared responses in the training study to the responses from a sample with no training in Study 1 (see Data Analysis section for full details) to estimate baseline responses.

The games were described in purely economic terms, did not mention “compassion” or “reappraisal”, participants were not instructed to use trainings, and the experimenter left the room during all economic decisions. All participants were administered the redistribution game first, and these results are previously reported in [3]. The altruistic punishment and helping games were administered in randomized order after the redistribution game, and participants did not play with live dictators. In each game, they witnessed a pre-programmed unfair (< 25%; Helping Game offer = 10%; Punishment Game offer = 15%) as well as a fair/generous dictator offer (50 or 60%) and were told they were playing with live players who were located in a different building. Fair/generous offers were only included to increase believability that participants were playing with other live players. Participants were debriefed after the experiment, and only those who believed they were interacting with live players were included for data analysis.

#### Data analysis

Third-party percentage scores were computed for the Helping and Punishment games. See data in S2 Dataset. The denominator used to compute punishment percentages accounted for the amount of the dictator offer (100–15). Percentage data were transformed into ranks for all games because of a non-normal distribution and the presence of outliers (> 3 SD from the population mean) in the redistribution game [3]. Differences between the Compassion and Reappraisal Training groups were tested with an independent *t*-test on the behavior ranks.

#### Determining whether Compassion Training changes altruistic behavior compared to the No Training Group

Because altruistic behavior was only measured after training, it is unclear whether group differences would indicate an increase and/or decrease compared to baseline behavior. Although baseline behavior was not measured, responses from the game participants who did not go through training can be used to estimate pre-training behavior (No Training group). As previously described in [3], Compassion and Reappraisal Training group means were compared to the No Training group mean by ranking third-party percentages across all 3 groups in each game (Punishment N = 130, Helping N = 119).

In each game, statistics were performed on the new ranks that compared 1) Compassion vs. No Training to determine whether Compassion Training increased altruistic behavior compared to a sample with no training, 2) Reappraisal vs. No Training to determine whether Reappraisal Training impacted altruistic behavior compared to a sample with no training, and 3) Compassion vs. Reappraisal Training using the new ranks to confirm the original *t*-test results. In the punishment game, the impact of social desirability was also accounted for using a hierarchical linear regression model due to the significant effect in the No Training group (Table 2). The main effect of social desirability and the interaction of Group × Social Desirability were entered into the first step, and the Group variable was entered into the second step to test the difference between Training (Compassion or Reappraisal) and No Training group. An independent *t*-test was used to test the difference between Compassion and Reappraisal Training groups on the new ranks.

Other significant confounding variables from the No Training group (e.g., transfer as the dictator in the helping game, see Table 2) were specific to the protocol design of having participants play in each role, which was not a design element in the Training protocol. Therefore, these variables were not taken into account when comparing Training and No Training groups. In the helping game, no relevant confounding variables were identified, so independent *t*-tests were used to test the difference between Training and No Training groups.

### Results

**Fig 3:**
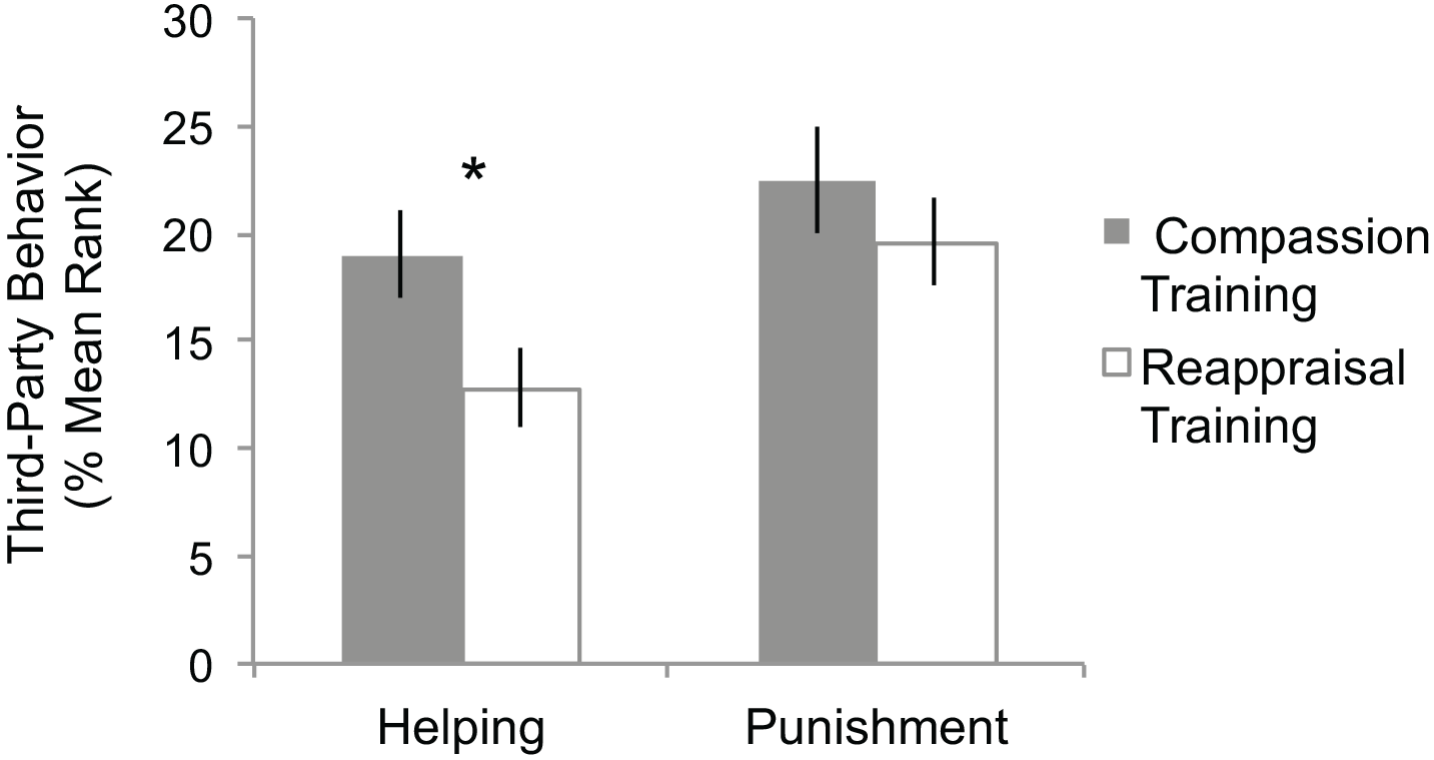
Altruistic behavior after short-term online Compassion Training or Reappraisal Training. Individuals who learn Compassion Training were more altruistic towards the Recipient in the Helping Game after witnessing an unfair interaction compared to Reappraisal Training (responses were ranked among 30 participants, * independent *t*_28_ = 2.29, *p* < .05). Compassion trainees spent 1.87 times more money than Reappraisal trainees in order to altruistically help the recipient. Compassion trainees on average spent $1.14 (mean rank = 19.0) and increased equality between the dictator by 29%, where the Reappraisal trainees spent an average of $0.61 (mean rank = 12.8) and increased the equality by 15%. Compassion Training did not significantly alter altruistic punishment towards the Dictator compared to Reappraisal Training. Punishment Game responses were ranked among 41 participants. Error bars indicate the standard error of the % mean rank.

After only two weeks of training, individuals who practiced Compassion Training were more willing to altruistically help (Compassion mean rank = 19.0 or $1.14, Reappraisal mean rank = 12.8 or $0.61, *t*_28_ = 2.29, *p* < 0.05) compared to those who practiced Reappraisal Training (Fig 3). In the Helping Game, compassion trainees spent 1.87 times more money than reappraisal trainees in order to altruistically help the recipient. Participants trained in compassion increased equality between the dictator and recipient by 29%, whereas those trained in reappraisal only increased the equality by 15%. Compassion Training did not influence behavior in the Punishment Game compared to Reappraisal Training (Fig 3; *t*_39_ = 0.91, *p* = 0.37).

In order to estimate whether compassion training may have increased helping and punishment behaviors compared to a pre-training level, training group means were compared to the means of the participants who did not go through training (No Training group). In the Helping Game, the Compassion Training group gave a similar amount compared to the No Training group (Compassion mean rank = 70.15, No Training mean rank = 61.49, *t*_1,100_ = 0.91, *p* = 0.36), whereas the Reappraisal Training group gave significantly less than the No Training group (Reappraisal mean rank = 44.41, No Training mean rank = 61.49, *t*_1,104_ = -2.00, *p* < 0.05). The Compassion Training group was still found to be more altruistic than the Reappraisal Training group in the Helping Game when comparing the newly computed ranks derived from combining the samples from Studies 1 and 2 (*p* < 0.05). In the Punishment Game (full sample and Punishers only), no group differed in punishment behavior from any other group (all *p*’s > 0.26).

## General Discussion

This study demonstrated that after two weeks of training, compassion training resulted in more altruistic helping of an anonymous victim but not punishment of a transgressor compared to reappraisal training. This suggests that a previous finding that compassion training increases altruistic redistribution [3], which is a combination of altruistic helping and punishment, is driven more by the helping than the punishment component of redistribution. These findings are supported by a previous study that demonstrated that long-term practitioners of compassion meditation spent more to recompensate a victim but did not differ from controls in how much they punished a transgressor who was unfair to another player [35].

However, these findings are not completely clear because when comparing responses in the Helping Game to a sample with no training, it appears that the difference between the compassion and reappraisal groups are driven by reappraisal training *decreasing* helping behavior rather than compassion training increasing it. This result should be considered preliminary because the altruistic helping paradigm was added later to the training protocol, and the N was lower compared to the punishment game (Helping N = 30, Punishment N = 41). In addition, helping and punishment games were administered after the redistribution game, and results may be more clean if the games are administered independently of each other. A limitation of the study is that the economic games were only administered post-training, and although we accounted for this by comparing training group responses to samples with no training, future studies can clarify the directionality of helping behavior through compassion training by administering games both pre- and post-training and having sufficient power to detect differences.

In this study, it appears that compassion training has no impact on punishment behavior. Further research is needed to determine whether compassion training would mitigate punishment behavior in Punishers, as shown in the sample with no training from Study 1 (the Punisher sample size was not large enough within the training sample to fully address this question). Although altruistic punishment is a behavior that appears to help enforce social norms and protect the well-being of other group members, these data suggest that compassion training may not have a direct influence on third-party punishment. To clarify the impact of compassion training on altruistic behavior from pre- to post-training, future studies should use paradigms that are validated for longitudinal study designs [2] or test altruistic behavior using multiple trials of varying offers [16,35].

It is also unclear what component(s) of the compassion training may be impacting altruistic behavior, and how much the qualities being trained in Study 2 are similar to or different from what trait empathic concern is measuring in Study 1. In the compassion training study, participants practiced enhancing both the emotional and motivational components of compassion, as well as learned to have a more balanced response to others’ suffering. It is unclear which of these components (or all) were tied to the changes in altruistic helping or redistribution behavior. In the samples with no training, we only assessed the emotional component of compassion (empathic concern) to predict altruistic behavior, and future studies should assess additional components of compassion. It is possible that the qualities impacted by compassion training are not necessarily the same construct that trait empathic concern is assessing in the general population. This issue can be aided by developing compassion questionnaires that are conceptualized from a Buddhist contemplative framework, and to use additional measures that may be more sensitive to detecting these different constructs such as psychophysiology or neuroimaging. In addition, although compassion training is likely increasing the tendency to respond with caring and wanting to help those who are suffering, because the training is only two weeks long, it is unlikely that this tendency would be sustained with time without continued practice. Future research should examine the sustained effect of compassion training on altruistic behavior through longitudinal studies.

Finally, it is worth noting that the intention of these studies is not to determine the “correct” way to behaviorally express a compassionate motivation, but rather to identify general patterns in the data that will help match compassion trainings to relevant causes and populations. For example, if compassion training specifically targets altruistic helping rather than punishment, it would be particularly relevant to implement compassion training to professionals who engage in helping those who are suffering (e.g., doctors and psychotherapists). Further research is needed to determine whether compassion training may be useful for professions that include sanctions such as law enforcement. According to contemplative traditions, the intention behind the action is as critical as the action itself [10]. From this perspective, punishment could arise from a compassionate motivation as much as helping behavior, and more nuanced experimental designs are needed to measure punishment that may arise from a compassionate stance. Our data from Study 1 suggest that compassion can decrease the amount of punishment given for those who choose to punish. Punishment decisions are contextual and involve integrating a number of factors including the intent of the transgressor and the severity of harm [15], and adopting a compassionate view towards those who violate societal norms may be beneficial for identifying optimal ways to rehabilitate offenders. More research is needed to examine the impact of compassion training on altruistic acts within tightly-controlled experimental paradigms as well as more complex real-world scenarios. Interventions can therefore be targeted to specific populations and contexts in order to improve interactions and social health in our increasingly diverse and interconnected society.

## Supporting Information

S1 Dataset Dataset from Study 1: Individual differences in empathic concern and altruistic behavior. (XLSX)

S2 Dataset Dataset from Study 2: Compassion Training Study. (XLSX)
